# PiRNA hsa_piR_019949 promotes chondrocyte anabolic metabolism by inhibiting the expression of lncRNA NEAT1

**DOI:** 10.1186/s13018-023-04511-z

**Published:** 2024-01-04

**Authors:** Xinhai Zhang, Xuyi Wang, Fengbin Yu, Chenglong Wang, Jianping Peng, Chuandong Wang, Xiaodong Chen

**Affiliations:** 1 Department of Orthopedic Surgery, Xinhua Hospital Affiliated to Shanghai Jiao Tong University School of Medicine (SJTUSM), Shanghai, China; 2 Department of Orthopaedics, The First Affiliated Hospital of Bengbu Medical College, Bengbu, China; 3 Department of Orthopaedics, The 72, Group Army Hospital of PLA, Huzhou, 313000 Zhejiang China

**Keywords:** Osteoarthritis, piRNA, Chondrocyte, NEAT1, NLRP3

## Abstract

**Background:**

Osteoarthritis is a prevalent degenerative joint condition typically found in individuals who are aged 50 years or older. In this study, the focus is on PIWI-interacting RNA (piRNA), which belongs to a category of small non-coding RNAs. These piRNAs play a role in the regulation of gene expression and the preservation of genomic stability. The main objective of this research is to examine the expression of a specific piRNA called hsa_piR_019949 in individuals with osteoarthritis, to understand its impact on chondrocyte metabolism within this condition.

**Methods:**

We analyzed piRNA expression in osteoarthritis cartilage using the GEO database. To understand the impact of inflammatory factors on piRNA expression in chondrocytes, we conducted RT-qPCR experiments. We also investigated the effect of piRNA hsa_piR_019949 on chondrocyte proliferation using CCK-8 and clone formation assays. Furthermore, we assessed the influence of piRNA hsa_piR_019949 on chondrocyte apoptosis by conducting flow cytometry analysis. Additionally, we examined the differences in cartilage matrix composition through safranine O staining and explored the downstream regulatory mechanisms of piRNA using transcriptome sequencing. Lentiviral transfection of NEAT1 and NLRP3 was performed to regulate the metabolism of chondrocytes.

**Results:**

Using RNA sequencing technology, we compared the gene expression profiles of 5 patients with osteoarthritis to 3 normal controls. We found a gene called hsa_piR_019949 that showed differential expression between the two groups. Specifically, hsa_piR_019949 was downregulated in chondrocytes when stimulated by IL-1β, an inflammatory molecule. In further investigations, we discovered that overexpression of hsa_piR_019949 in vitro led to increased proliferation and synthesis of the extracellular matrix in chondrocytes, which are cells responsible for cartilage formation. Conversely, suppressing hsa_piR_019949 expression resulted in increased apoptosis (cell death) and degradation of the extracellular matrix in chondrocytes. Additionally, we found that the NOD-like receptor signaling pathway is linked to the low expression of hsa_piR_019949 in a specific chondrocyte cell line called C28/I2. Furthermore, we observed that hsa_piR_019949 can inhibit the expression of a long non-coding RNA called NEAT1 in chondrocytes. We hypothesize that NEAT1 may serve as a downstream target gene regulated by hsa_piR_019949, potentially influencing chondrocyte metabolism and function in the context of osteoarthritis.

**Conclusions:**

PiRNA hsa_piR_019949 has shown potential in promoting the proliferation of chondrocytes and facilitating the synthesis of extracellular matrix in individuals with osteoarthritis. This is achieved by inhibiting the expression of a long non-coding RNA called NEAT1. The implication is that by using hsa_piR_019949 mimics, which are synthetic versions of the piRNA, as a therapeutic approach, it may be possible to effectively treat osteoarthritis.

**Supplementary Information:**

The online version contains supplementary material available at 10.1186/s13018-023-04511-z.

## Introduction

Osteoarthritis (OA) is a prevalent, long-lasting condition that affects the joints. It is characterized by the gradual deterioration and loss of articular cartilage, which results in pain, joint stiffness, and reduced functionality. This degenerative process can significantly impact a patient's overall quality of life [1]. Currently, the available treatment options for osteoarthritis (OA) are typically limited to invasive procedures such as joint replacement surgery and methods aimed at providing symptomatic relief [2]. Hence, it is crucial to explore novel disease mechanisms to enable the development of more precise therapies. Chondrocytes, the only type of cells present in articular cartilage, play key roles in responding to injuries, maintaining tissue stability, and participating in the process of cartilage reconstruction in osteoarthritis (OA). In normal physiological conditions, chondrocytes strike a dynamic equilibrium between differentiation and apoptosis, as well as the synthesis and degradation of the cartilage extracellular matrix. However, in the presence of OA, this equilibrium is disrupted, leading to the breakdown of the cartilage matrix and excessive chondrocyte apoptosis. Ultimately, this results in the degeneration of articular cartilage [3]. The regulation of extracellular matrix synthesis and degradation in chondrocytes is of utmost importance in maintaining the metabolic process of articular cartilage. The molecular mechanisms that control this delicate balance play a critical role in ensuring the overall health and functionality of the cartilage [4]. Identifying and targeting this particular aspect could prove crucial in therapeutic interventions aimed at treating osteoarthritis and effectively delaying the degeneration of cartilage.

PIWI-interacting RNA (piRNA) is a group of small RNA molecules that do not code for proteins. They typically have lengths ranging from 24 to 35 nucleotides. One of the distinguishing features of piRNA is the presence of a 2′-O-methylation modification at the 3' end. These piRNAs are specifically recognized and bound by PIWI proteins, which explains the name “PIWI-interacting RNA” [5, 6]. In the beginning, research indicated that piRNAs were predominantly found in the reproductive system. Their primary roles were believed to include safeguarding the genome by facilitating the breakdown of transcripts and regulating the structure of chromatin to inhibit the activity of transposons [7]. Advancements in high-throughput sequencing technologies have led to the discovery of approximately 20,000 piRNA genes within the human genome [8]. There is a growing body of evidence indicating that piRNAs, previously thought to be exclusive to germline cells, are also present in somatic cells. The disruption of piRNA expression and function has been linked to several health conditions, including cancer, reproductive disorders, neurodegenerative diseases, and the process of aging [9–11].

The current focus of research on piRNA revolves around its role in regulating cancer occurrence and development. Specific types of cancer, including colorectal cancer, breast cancer, and lung cancer, are particularly studied in this context. Moreover, recent studies also suggest that small interfering RNAs contribute to various human diseases such as osteoporosis, rheumatoid arthritis, tendon injuries, tendon homeostasis, and osteoarthritis [12–16]. To date, there have been no reported studies examining the relationship between piRNAs (PIWI-interacting RNAs) and osteoarthritis or cartilage. Additionally, the exact role of piRNAs in chondrocytes remains unclear. Further research is required to investigate the impact of piRNAs on cartilage metabolism in osteoarthritis and to elucidate the mechanisms through which piRNAs regulate long non-coding RNAs (lncRNAs) and activate inflammatory processes.

## Methods

### Bioinformatics analysis

Bioinformatics analysis was conducted using OmicStudio software (https://www.omicstudio.cn/tool). Volcano plots or other graphics were generated using R on the OmicStudio platform (https://www.r-project.org/https://www.omicstudio.cn/tool). A small RNA sequencing dataset (GSE143514) containing samples from 3 osteoarthritis (OA) patients and 5 normal controls was downloaded from the GEO database for piRNA expression analysis. The Cutadapt program was utilized to remove adapter sequences from the raw offline data. The Trimmomatic program was employed to discard low-quality sequences and obtain clean data. The Fastqc program was used to assess the data quality of the clean data, and the fragments larger than 15 nucleotides were retained for subsequent analysis. The clean data were aligned to the piRNA database and genome using the bowtie program. Differential expression analysis of piRNAs was performed using edger, and piRNAs with significant differences between the two comparison groups were selected based on a *P* value < 0.05 and fold change (FC) > 2 or FC < 0.5.

### Cell culture

The C28/I2 and SW1353 cell lines were maintained in Dulbecco’s modified Eagle medium (DMEM)/F-12 (Thermo Fisher, California, USA) with 10% fetal bovine serum (FBS, BIOEXPLORER, California, USA) at 37 °C in a humidified chamber with 5% CO_2_. For stimulation with inflammatory factors, the chondrocytes were cultured in six-well plates and treated with IL-1β (10 ng/mL), TNF-α (10 ng/mL), or lipopolysaccharide (LPS) (1 μg/mL) for 24 h.

### Cell transfection

The hsa_piR_019949 mimic, hsa_piR_019949 inhibitor, and their negative controls were supplied by General Bio-company (General Biol, Anhui, China). Cell transfection was conducted by using the Lipofectamine 2000 reagent kit (Thermo Fisher, California, America). The transfection dose of hsa_piR_019949 mimic and hsa_piR_019949 inhibitor was 50 nM, respectively. The following experiments were conducted 48 h after transfection.

### Real-time qPCR

Total RNA was extracted from cells using TRIzol reagent (Invitrogen, California, USA). For piRNA analysis, the piRNA was reverse transcribed into cDNA using the EZB-miRT2-plus-L kit (EZBioscience, Roseville, USA), and the relative expression level of piRNA was normalized to U6 controls. For mRNA analysis, the mRNA was reverse transcribed into cDNA using the HiScript III 1st Strand cDNA Synthesis Kit (Vazyme, Nanjing, China), and the relative expression level of genes was normalized to the internal control GAPDH. Real-time quantitative polymerase chain reaction (qPCR) was performed on a LightCycler 480 instrument using the ChamQ Universal SYBR qPCR Master Mix kit (Vazyme, Nanjing, China), following the manufacturer's instructions. The 2 − ∆∆*Ct* method was used to calculate the relative expression of piRNA and mRNA. All qPCR experiments were conducted in triplicate using a LightCycler 480 System (Roche, Basel, Switzerland), and the primers were obtained from Sangon Biotech (Shanghai, China) Co., Ltd. The sequence information of hsa_piR_019949 is as follows: 5′-GCCUGGAUAGCUCAGUUGGUAGAGCAUCAGA-3′. The sequence information of primers are listed in Additional file 1: Table S1.

### Flow cytometry

The analysis to determine C28/I2 cell apoptosis was performed using the Annexin V-FITC Apoptosis Detection kit (Beyotime, Shanghai, China). C28/I2 cells were digested with trypsin without EDTA, and 3 mL of cell suspension was transferred into 10 mL centrifuge tubes and centrifuged at 500 rpm for 5 min. After removing the culture medium, the cells were washed with PBS and centrifuged again at 500 rpm for 5 min. The supernatant was discarded, and the cells were resuspended in 100 μL of binding buffer. Then, 5 μL of Annexin V-FITC and 5 μL of propidium iodide (PI) were added and gently mixed. The mixture was incubated in the dark at room temperature for 15 min. Flow cytometry analysis was performed using a BD Aria flow III cytometer (BD, New Jersey, USA) to detect the fluorescence of FITC and PI and calculate the apoptotic rate. The flow cytometry results were analyzed using Flow Jo software v10.

### Clone formation analysis

In this study, the clone formation analysis method was used to assess the ability of cells to form colonies. Briefly, C28/I2 cells were seeded in 6-well plates at a density of 200 cells per well. After 24 h of incubation, the cells were treated with the experimental conditions. The medium was changed every three days to maintain cell viability. After 14 days of incubation, the cells were fixed with 4% paraformaldehyde for 15 min and stained with crystal violet for 30 min. Excess stain was washed off with water, and the plates were air-dried. The number of colonies containing at least 50 cells was counted under a microscope. The clone formation efficiency (CFE) was calculated as the ratio of the number of colonies formed to the number of cells seeded, multiplied by 100%. This analysis was performed in triplicate for each experimental condition. Statistical analysis was carried out using appropriate tests to determine significant differences between groups.

### CCK-8 analysis

In this study, the Cell Counting Kit-8 (CCK-8) assay was used to evaluate cell viability and proliferation. C28/I2 cells were seeded in a 96-well plate at a density of 2,000 cells per well. After 0, 12, 24, 48, 72, and 96 h, 10 μL of CCK-8 solution was added to each well, and the plate was incubated for an additional 2 h at 37 °C. The absorbance was measured at 450 nm using a microplate reader. The cell viability was calculated by comparing the absorbance of the treated cells to that of the control cells. The assay was performed in triplicate for each experimental condition, and the results were expressed as the mean ± standard deviation. Statistical analysis was conducted to determine significant differences between groups using appropriate tests.

### Immunofluorescence staining

In this study, the immunofluorescence staining technique was utilized to investigate the expression of Collagen II and MMP13 in chondrocytes. To initiate immunofluorescence staining, the sample was first fixed using a stationary solution and then permeabilized with Triton X-100. A blocking buffer containing bovine serum albumin (BSA) was employed to prevent nonspecific binding. Following that, primary antibodies specific to Collagen II and MMP13 were added to the sample and left to incubate overnight at a temperature of 4 °C. Subsequently, the sample was washed multiple times with phosphate-buffered saline (PBS) to remove any unbound antibodies. The second antibody, which was Cy3 conjugated with a fluorophore, was then introduced to the sample and incubated at room temperature for 1 h. After thorough washing, the sample was fixed onto a glass slide using an anti-fading fixing medium that included 4', 6-diamidino-2-phenylindole (DAPI) for nuclear staining. Finally, the glass slide was examined under a fluorescence microscope, and an image was captured using a filter set suitable for each fluorophore. The fluorescence signal was quantified and analyzed using image analysis software.

### Safranin O staining

C28/I2 cells were seeded in 6-well plates at a density of 20,000/well. After reaching approximately 70–80% confluency, the cells were fixed with 4% paraformaldehyde for 15 min at room temperature. Cells were then washed with phosphate-buffered saline (PBS) and stained with Safranin O solution (0.1% Safranin O in distilled water) for 10 min at room temperature. Excess stain was removed by washing with distilled water, and the stained cells were visualized using a brightfield microscope. Images were captured, and the intensity of Safranin O staining was quantified using image analysis software.

### Virus transduction

Insert the target gene or RNA sequence into the appropriate site of the slow virus vector. Cultivate and amplify the C28/I2 cell line. Once the cell density reaches an appropriate level, add polyethyleneimine or polyvinyl alcohol to transduce the slow virus vector into the cells. After transduction, treat the cells appropriately to facilitate the integration and expression of the slow virus.

### Statistical analysis

All experiments in this study were performed in triplicate and showed consistent results. The data were presented as mean ± standard deviation (SD). Statistical analysis was performed using the unpaired two-tailed Student's t-test for comparisons between two groups, and one-way analysis of variance (ANOVA) or two-way ANOVA followed by Tukey post hoc test for comparisons among multiple groups. A *P* value less than 0.05 was considered statistically significant. GraphPad Prism software (Version 6.01) was used for all statistical analyses. The differentially expressed genes were identified based on the criteria of fold change ≥ 2 and *P* value ≤ 0.05, as determined by bioinformatics analysis and mRNA sequencing results.

## Result

### PiRNA hsa_piR_019949 is downregulated in chondrocytes stimulated by inflammation

**Fig. 1:**
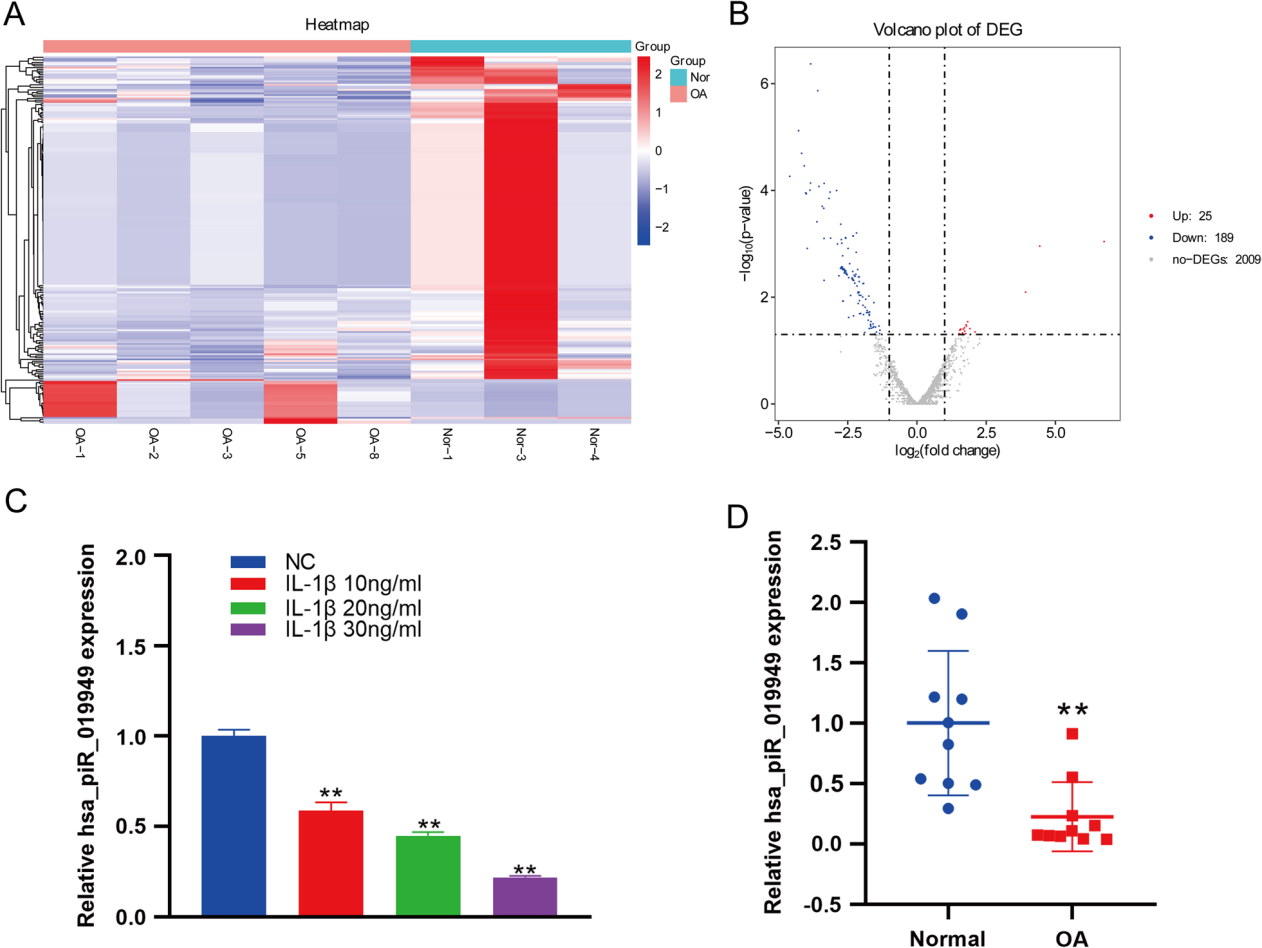
illustrates the downregulation of the piRNA hsa_piR_019949 in osteoarthritis. **A** The heatmap depicts the variation in expression levels of piRNAs between cartilage affected by osteoarthritis (OA) and healthy, normal cartilage. **B** The volcano map illustrates the differential expression patterns of piRNAs in cartilage affected by osteoarthritis (OA) compared to normal cartilage. **C** The expression of hsa_piR_019949 in C28/I2 cells, stimulated with IL-1β at concentrations of 10 ng/ml, 20 ng/ml, and 30 ng/ml, was detected using RT-qPCR. **D** The expression of hsa_piR_019949 was analyzed using RT-qPCR in both OA (osteoarthritis) and normal tissues. Statistically significant differences are indicated by *P < 0.05, **P < 0.01. *n* = 3/group; piRNAs = piwi-interacting RNAs; OA = osteoarthritis; NC = negative control; RT-qPCR = real-time quantitative PCR

By comparing the RNA expression profiles of knee chondrocytes in patients with osteoarthritis (OA) and normal controls, we identified 214 genes that are differentially expressed in the OA environment (Fig. 1A). Out of these genes, 25 were significantly upregulated, and 189 were significantly downregulated. Among the differentially expressed piRNAs, we discovered a small RNA called piRNA hsa_piR_019949, which exhibited the greatest reduction in expression in C28/I2 cells under IL-1β stimulation (Fig. 1B and Additional file 2: Fig. S1). To confirm our findings, we analyzed the C28/I2 cell line’s RNA after stimulating it with the inflammatory factor IL-1β at varying concentrations (10 ng/mL, 20 ng/mL, 30 ng/mL). Following stimulation, we observed a notable downregulation of piRNA hsa_piR_019949, and its expression level exhibited an inverse relationship with the inflammatory factor concentration (Fig. 1C). Additionally, we collected cartilage samples from 10 patients undergoing knee replacement surgery. For the osteoarthritis group, the cartilage tissue was obtained from weight-bearing areas of the joints, while for the normal control group, it was taken from non-worn areas. Upon extracting total RNA, we found a significant downregulation of hsa_piR_019949 expression in human knee arthritis tissues (Fig. 1D). These results provide more evidence for the potential contribution of piRNA hsa_piR_019949 to the occurrence of osteoarthritis and its role in inflammatory regulation.

### Overexpression of hsa_piR_019949 can promote the anabolism and inhibit catabolism of chondrocytes in vitro

**Fig. 2:**
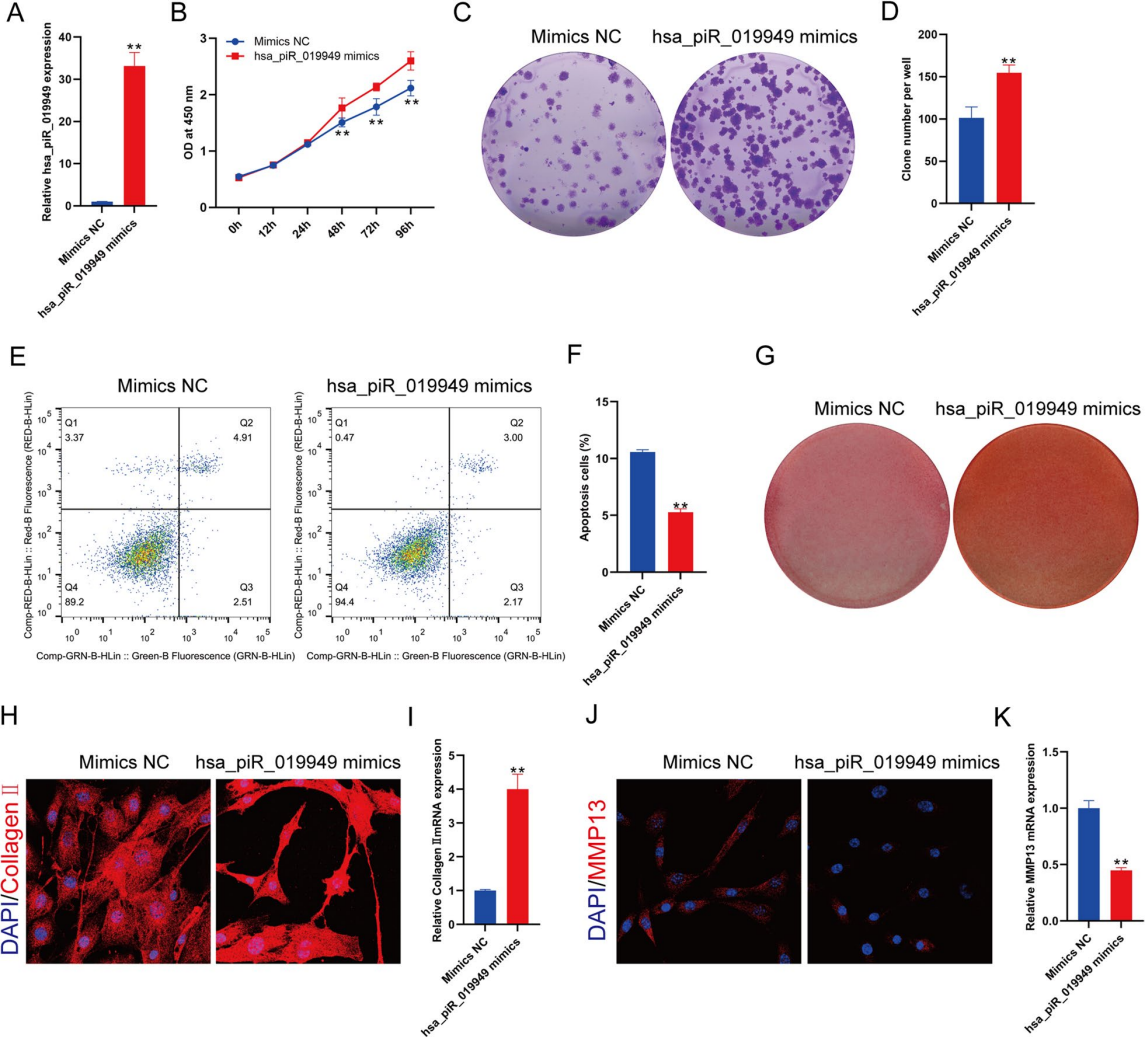
The overexpression of piRNA hsa_piR_019949 has been found to promote the anabolic metabolism and inhibited catabolic of chondrocytes. **A** The expression of hsa_piR_019949 in chondrocytes transfected with hsa_piR_019949 mimics is being studied. **B** The detection of chondrocytes transfected with hsa_piR_019949 mimics was accomplished using CCK-8 assays. **C** The ability of chondrocytes transfected with hsa_piR_019949 mimics to form clones was detected using crystal violet staining. **D** Quantitative analysis of the clone formation number of chondrocytes. **E** The flow cytometry technique was employed to detect the apoptosis of chondrocytes that were transfected with hsa_piR_019949 mimics. **F** Quantitative analysis of the normal cells of chondrocyte detected by flow cytometry. (**G**) Safranine O was used to quantify the cartilage extracellular matrix. The protein expression of collagen II (**H**) and MMP13 (**J**) in chondrocytes transfected with hsa_piR_019949 mimics was detected through immunofluorescence staining. The mRNA expression of collagen II (**I**) and MMP13 (**K**) in chondrocytes transfected with hsa_piR_019949 mimics was detected using RT-qPCR. Statistically significant differences are indicated by *P < 0.05, **P < 0.01. *n* = 3/group

To validate the function of piRNA hsa_piR_019949, we carried out number of experiments. Firstly, we created a cellular model that overexpressed piRNA hsa_piR_019949 by designing a mimic of the piRNA and transfected it into the C28/I2 chondrocyte cell line (Fig. 2A). Our CCK-8 assay results indicated that the overexpression of piRNA hsa_piR_019949 significantly enhanced cell proliferation in the chondrocyte cell line after 48 h (Fig. 2B). Similarly, the colony formation assay demonstrated consistent findings (Fig. 2C, D). We labeled cells with apoptosis-specific fluorescent markers and employed flow cytometry to assess cell apoptosis rate. Our analysis revealed that the chondrocyte cell line transfected with the piRNA hsa_piR_019949 mimic exhibited a significant reduction in apoptosis compared to the control (Fig. 2E), thus indicating an increase in viable cells (Fig. 2F). Furthermore, Safranin O staining was employed to examine the impact of piRNA hsa_piR_019949 on chondrocytes. The results indicated that chondrocytes transfected with the piRNA hsa_piR_019949 mimic exhibited darker staining, which suggests the presence of a greater amount of cartilage matrix (Fig. 2G and Additional file 3: Fig. S2A). By utilizing RT-qPCR and immunofluorescence protein verification, we found that transfection with the piRNA hsa_piR_019949 mimic resulted in a significant increase in the transcription and protein levels of Collagen II (Fig. 2H, I). Additionally, it was found that the expression of MMP13 had decreased (Fig. 2J, K).

### Knockdown of hsa_piR_019949 can inhibit the anabolism and promote catabolism of chondrocytes in vitro

**Fig. 3:**
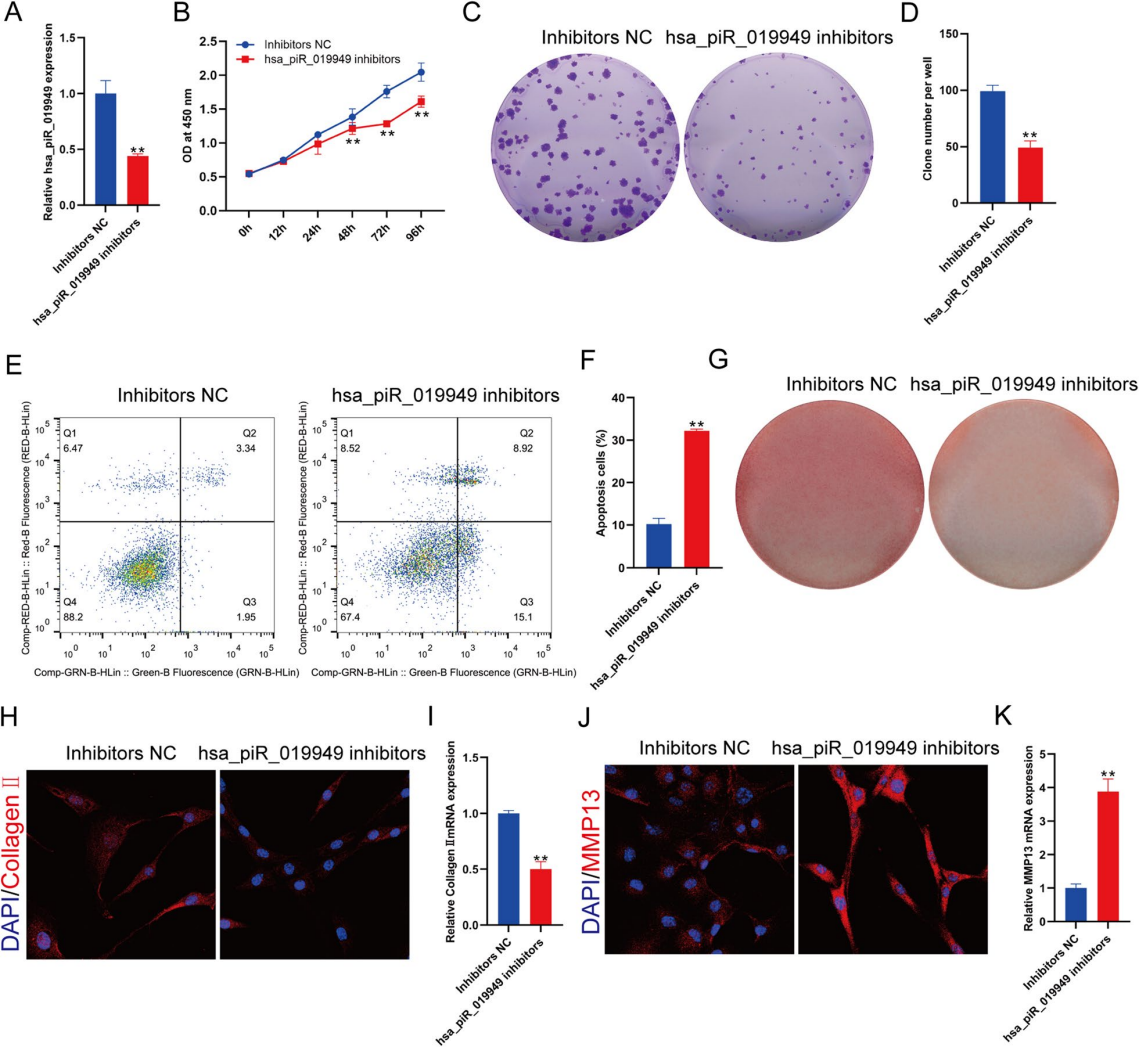
Knockdown of piRNA hsa_piR_019949 leads to a decrease in the anabolic metabolism and increase in the catabolic metabolism of chondrocytes. **A** The study investigated the effect of inhibiting hsa_piR_019949 on chondrocytes. **B** The expression of hsa_piR_019949 in chondrocytes was examined after transfection with an inhibitor. Cell proliferation was assessed using CCK-8 assays. **C** The ability of chondrocytes to form clones was evaluated through crystal violet staining. **D** The number of clones formed by chondrocytes was quantitatively analyzed. **E** The apoptosis of chondrocytes transfected with the inhibitor was measured using flow cytometry, **F** specifically looking at the percentage of normal cells. **G** The quantification of cartilage extracellular matrix was determined using safranine O staining. The protein expression of collagen II (**H**) and MMP13 (**J**) in chondrocytes transfected with hsa_piR_019949 mimics was detected through immunofluorescence staining. The mRNA expression of collagen II (**I**) and MMP13 (**K**) in chondrocytes transfected with hsa_piR_019949 mimics was detected using RT-qPCR. Statistically significant differences are indicated by *P < 0.05, **P < 0.01. *n* = 3/group

In our study, we created a model of the human normal chondrocyte cell line with suppressed expression of hsa_piR_019949 by developing an inhibitor for hsa_piR_019949 (Fig. 3A). The results obtained from CCK-8 and clone proliferation experiments demonstrated that the inhibition of hsa_piR_019949 expression led to a decrease in the proliferation of the chondrocyte cell line (Fig. 3B–D). Furthermore, the analysis of apoptosis using flow cytometry revealed that the suppression of hsa_piR_019949 expression promoted cell death in an inflammatory environment (Fig. 3E, F. Staining with Safranin O showed a lighter color after transfecting the chondrocyte cell line with the hsa_piR_019949 inhibitor, indicating a reduction in the cartilage matrix (Fig. 3G and Additional file 3: Fig. S2B). Furthermore, the expression of Collagen II decreased upon inhibition of hsa_piR_019949 (Fig. 3H, I), whereas the expression of MMP-13 increased (Fig. 3J, K).

### NOD-like receptor signaling pathway is correlated with high expression of hsa_piR_019949 in C28/I2

**Fig. 4:**
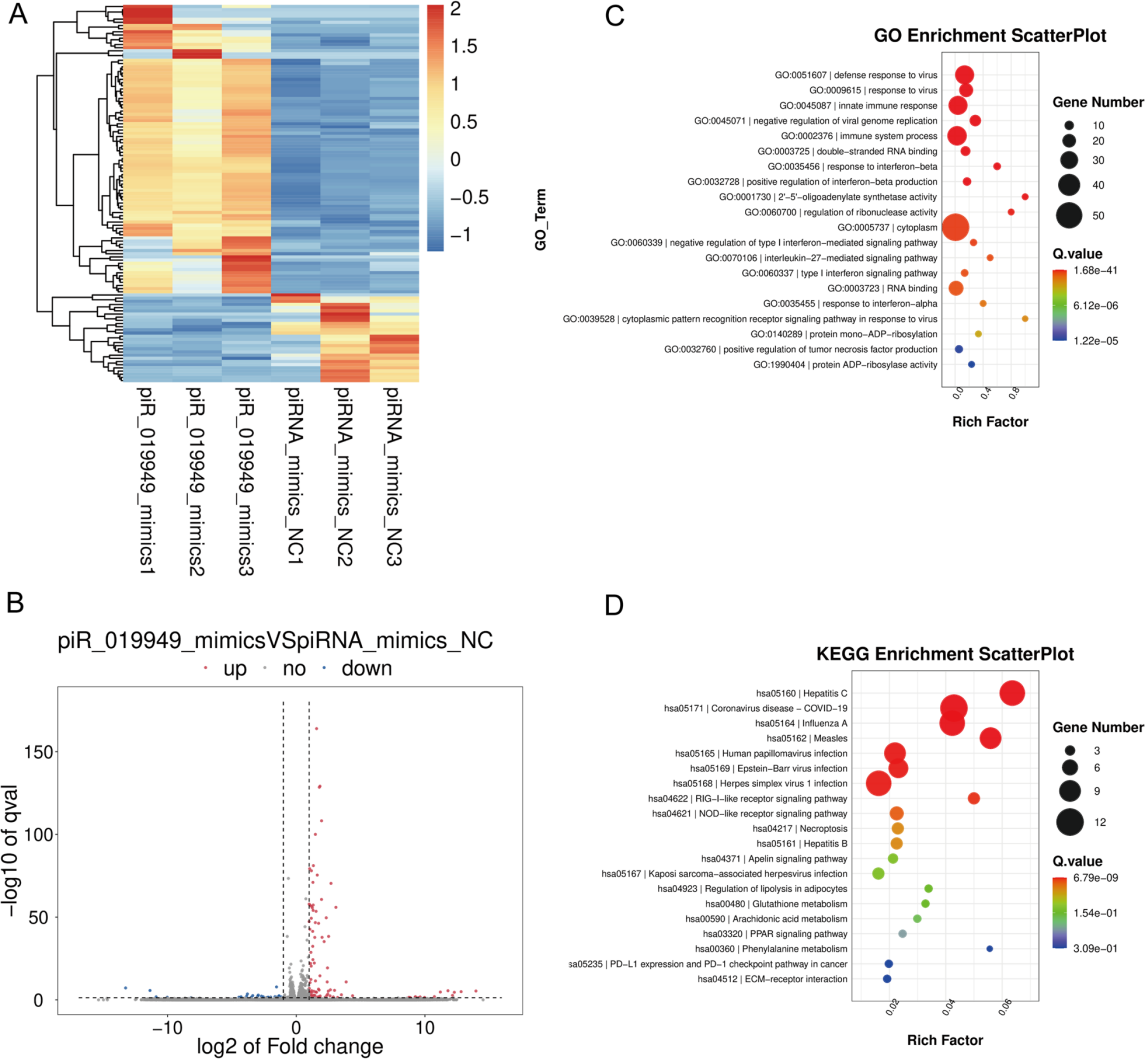
The NOD-like receptor signaling pathway is associated with high expression levels of hsa_piR_019949 in C28/I2 cells. **A** The heatmap displays the contrasting levels of piRNAs that are expressed in C28/I2 cells transfected with hsa_piR_019949 mimics in comparison to the normal control. **B** The volcano map illustrates the differential expression of piRNAs in C28/I2 cells transfected with hsa_piR_019949 mimics compared to the normal control. **C** The GO enrichment analysis highlights the enrichment of differentially expressed mRNA in terms of Gene Ontology categories. **D** The KEGG enrichment analysis reveals the enrichment of differentially expressed mRNA in relation to the Kyoto Encyclopedia of Genes and Genomes (KEGG) pathways

To investigate the regulatory mechanism of hsa_piR_019949 in C28/I2 metabolism, we conducted RNA sequencing analysis on three samples transfected with hsa_piR_019949 mimic and three control groups. Through differential gene expression analysis, we generated volcano plots and heatmaps to visualize the differences in gene expression. Notably, we found that 19,754 genes were upregulated, while 19,128 genes including the lncRNA NEAT1 and NLRP3 were downregulated (Fig. 4A, B). Subsequent GO (Gene Ontology) and KEGG (Kyoto Encyclopedia of Genes and Genomes) analyses enabled the identification of potential downstream signaling pathways influenced by hsa_piR_019949. Several biological functions related to defense against viruses, immune system processes, and signaling pathways such as Human papillomavirus infection, RIG-I-like receptor signaling pathway, and NOD-like receptor signaling pathway were found to be associated with the expression of hsa_piR_019949. Previous studies have suggested that the NOD-like receptor signaling pathway is linked to the activation of the inflammasome protein NLRP3 [17] (Fig. 4C, D). Hence, it is plausible that hsa_piR_019949 may impact C28/I2 metabolism by modulating the NOD-like receptor signaling pathway. This novel finding provides valuable insights for exploring the functions and mechanisms of hsa_piR_019949 in the future.

### LncRNA NEAT1 serves as a downstream target gene regulated by hsa_piR_019949 in chondrocyte metabolism

**Fig. 5:**
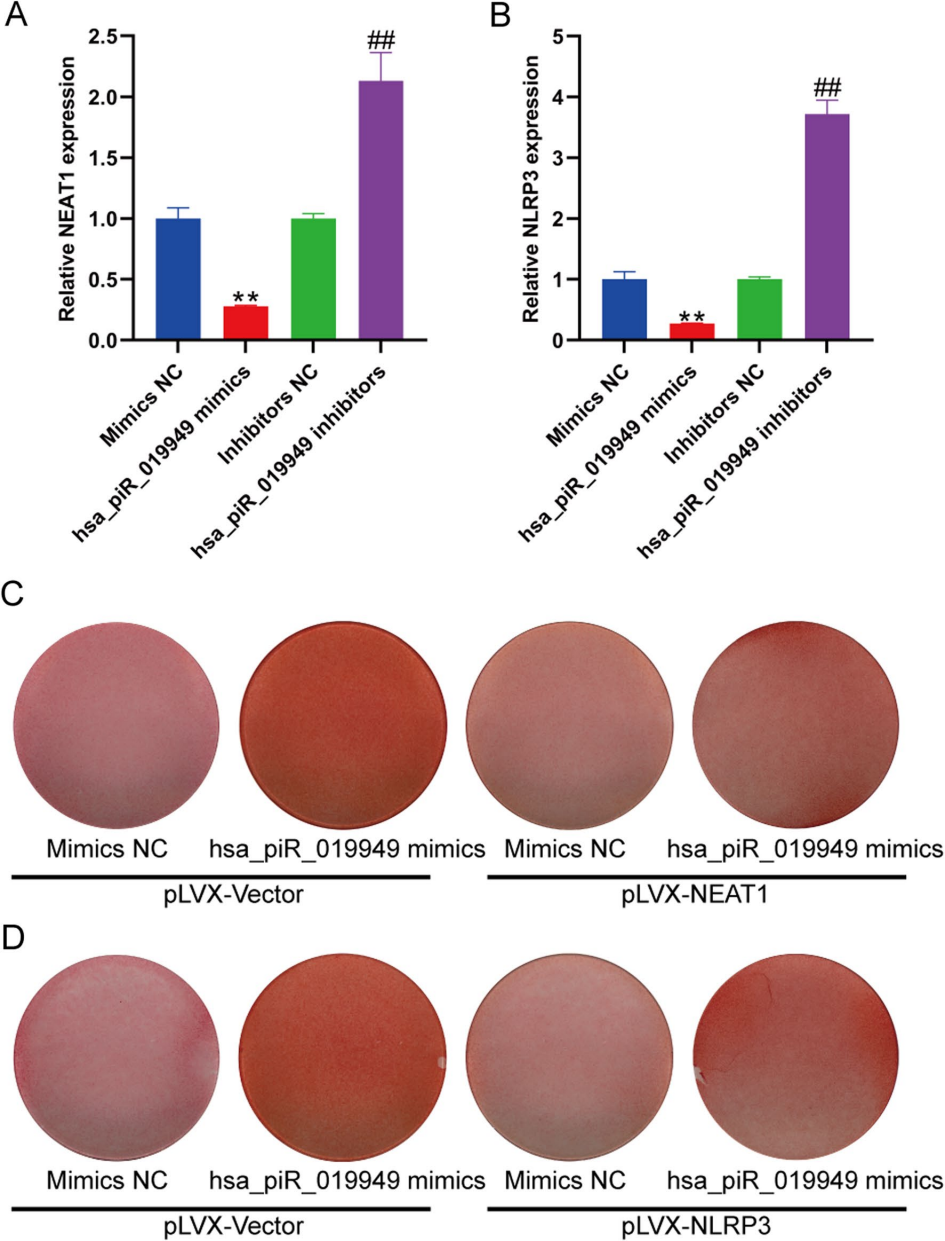
Long non-coding RNA NEAT1 acts as a gene that is influenced by hsa_piR_019949 in the regulation of chondrocyte metabolism. **A** The mRNA expression of lncRNA NEAT1 in chondrocytes transfected with hsa_piR_019949 mimics and inhibitor were detected by RT-qPCR. **B** The expression of NLRP3 in chondrocytes transfected with hsa_piR_019949 mimics and inhibitor were detected by RT-qPCR. **C** The quantification of cartilage extracellular matrix after transfected with NEAT1 was detected by Safranine O. **D** The quantification of cartilage extracellular matrix after transfected with NLRP3 was detected by Safranine O

The expression levels of lncRNA NEAT1 were validated to be downregulated following transfection with hsa_piR_019949 mimic, whereas the inhibitor group showed upregulated expression using RT-qPCR (Fig. 5A). Additionally, the downregulation of NLRP3, a key gene in the NOD-like receptor signaling pathway, was confirmed with elevated expression of hsa_piR_019949 (Fig. 5B). Following transfection with hsa_piR_019949 mimic, lentivirus-mediated transfection of NEAT1 (Additional file 4: Fig. S3) and NLRP3 was then performed separately in both control and experimental groups. By comparing the cartilage composition using Safranin O staining, it was observed that the promoting effect of hsa_piR_019949 on chondrocyte synthesis metabolism was suppressed by NEAT1 and NLRP3. This suggests that NEAT1 and NLRP3 may function as downstream targets of hsa_piR_019949 (Fig. 5C, D and Additional file 5: Fig. S4).

## Discussion

In summary, the piRNA called hsa_piR_019949 is downregulated in chondrocytes when inflammation occurs. A comparison of RNA expression profiles in knee chondrocytes between patients with osteoarthritis (OA) and normal controls identified 214 genes that showed changes. Among these, 25 genes were significantly upregulated, and 189 genes were significantly downregulated. One of the small RNAs found was hsa_piR_019949. Further validation demonstrated that the expression level of hsa_piR_019949 decreased significantly under the stimulation of inflammatory factors and showed an inverse correlation with the concentration of these factors. Moreover, hsa_piR_019949 was also found to be significantly downregulated in the articular cartilage of OA patients, reinforcing its potential role in the development of OA and regulation of inflammation.

Further research revealed that hsa_piR_019949 is involved in the functional regulation of chondrocytes in vitro by influencing chondrocyte synthesis and degradation. Overexpression of hsa_piR_019949 promoted chondrocyte proliferation, reduced apoptosis, and increased cartilage matrix synthesis. Conversely, inhibiting the expression of hsa_piR_019949 suppressed chondrocyte proliferation, promoted apoptosis, and facilitated degradation of the cartilage matrix. Additionally, hsa_piR_019949 is associated with the NOD-like receptor signaling pathway, impacting chondrocyte metabolism through the regulation of this signaling pathway.

Currently, the focus of research on non-coding RNA in osteoarthritis is primarily on microRNAs (miRNAs). Studies by Ito et al. [18] have shown that microRNA-455 (miR-455), specifically miR-455-5p and miR-455-3p, are highly expressed in human and mouse primary chondrocytes. These miRNAs up-regulate the key transcription factor Sox9. Another study by Yen-You Lin et al. [19] uncovered that miR-144-3p can directly bind to IL-1β, reducing its expression level and thus alleviating the damage it causes to cartilage. Additionally, miR-144-3p was found to slow down the progression of osteoarthritis in a rat model of anterior cruciate ligament and meniscus surgery.

NEAT1 is an RNA molecule that interacts with various RNA-binding proteins and plays a role in important biological processes such as RNA metabolism, transcriptional regulation, and cellular stress responses [20, 21]. It has also been found to be highly expressed in various tumors and is involved in tumor initiation and progression [22, 23]. NEAT1 regulates the expression and activity of NLRP3, an important component of the inflammasome involved in inflammation and immune regulation [24]. Overexpression of NEAT1 leads to excessive activation of NLRP3 and heightened inflammatory response [25]. NEAT1 interacts with NLRP3 mRNA, controlling its stability and translation. It also forms complexes with other RNA-binding proteins to regulate NLRP3 inflammasome formation and activation. The NEAT1-NLRP3 axis is implicated in various diseases, including inflammatory conditions like rheumatoid arthritis and inflammatory bowel disease, as well as neurodegenerative disorders such as Alzheimer's and Parkinson's diseases [26–29]. Understanding the relationship between NEAT1 and NLRP3 is crucial for understanding inflammatory responses and immune regulation. However, in osteoarthritis, NEAT1 and NLRP3 seem to have a relieving effect. The purpose of this study is to use hsa_piR_019949 to regulate the expression of NEAT1 and NLRP3 in order to reverse chondrocyte apoptosis and degradation of the extracellular matrix. It is also investigating whether lncRNA NEAT1 is a downstream target gene of hsa_piR_019949 in chondrocyte metabolism. Experimental results from simulated transfection of hsa_piR_019949 show that the expression levels of NEAT1 and NLRP3 were downregulated. Further experiments confirm that NEAT1 and NLRP3 inhibit the promotion of hsa_piR_019949 on chondrocyte anabolism. These findings suggest that NEAT1 and NLRP3 may indeed be the downstream target genes of hsa_piR_019949.

According to Xin, Liu et al. [30], the NLRP3 pathway has been shown to play a significant role in promoting oxidative stress and apoptosis in synovitis-induced stimulation. Specifically, the ALPK1/NF-κB signaling pathway disrupts the redox balance and increases the production of ROS by inducing cytokine secretion, including IL-1β and TNF-α. This, in turn, promotes the NLRP3/Caspase-1/GSDMD signaling pathway, leading to apoptosis. The study also suggests that intervention in the NLRP3 pathway can reduce oxidative stress and inflammatory damage. Shaocong, Li et al. [31] demonstrated that iron overload leads to the formation of NLRP3 inflammatory corpuscles, resulting in chondrocyte apoptosis and arthritis. Their study showed that Cardamonin (CAR) can inhibit NLRP3 by activating SIRT1 and inhibiting the expression of the p38MAPK signaling pathway. This intervention reduces chondrocyte apoptosis and arthritis caused by iron overload. Therefore, the NLRP3 pathway plays a role in inducing inflammatory reactions and chondrocyte apoptosis, and inhibiting NLRP3 can treat iron overload-induced arthritis using CAR. Xiaohang, Zheng et al. [32] believe that NLRP3 is involved in the occurrence and development of inflammatory reactions and oxidative damage in articular cartilage. NLRP3-mediated pyroptosis plays a crucial role in the pathological process of osteoarthritis, and the activation of the NF-κB pathway exacerbates the inflammatory response. The study investigated the effects of paroxetine, an antidepressant, on chondrocytes in articular cartilage. They measured the expression levels of NLRP3, IL-1β, CASP1, and other related proteins in ATDC5 cells and mouse models. The results showed that paroxetine treatment reduced the inflammatory response in articular cartilage by inhibiting the activation of the NF-κB signaling pathway and suppressing pyroptosis.

In summary, the study revealed that the piRNA hsa_piR_019949 may play a significant role in the development of osteoarthritis (OA) and the regulation of inflammation. It also demonstrated the functional regulation of this specific piRNA in the synthesis and degradation of chondrocytes. Furthermore, the study delved deeper into the relationship between hsa_piR_019949 and the NOD-like receptor signaling pathway, as well as NEAT1/NLRP3. These findings offer valuable insights for further investigation into the function and mechanism of hsa_piR_019949 with OA.

## Conclusion

In conclusion, our study indicates that focusing on the piRNA hsa_piR_019949 and suppressing the expression of lncRNA NEAT1 holds promise as a therapeutic strategy for osteoarthritis. This approach has the potential to stimulate the growth of chondrocytes and improve the production of extracellular matrix, which are crucial for the repair and regeneration of cartilage. Further investigation is necessary to comprehensively comprehend the underlying mechanisms and to establish the effectiveness and safety of hsa_piR_019949 mimics as a treatment for osteoarthritis.

### Supplementary Information

**Additional file 1**. **Table S1**: The primer sequence information.

**Additional file 2**. **Figure S1**: IL-1β regulated the expression of piRNA in C28/I2 cells.

**Additional file 3**. **Figure S2**: Quantification of Safranin O staining of C28/I2 cells with hsa_piR_019949 overexpression (A) or knockdown (B). **P < 0.01. n = 3/group.

**Additional file 4**. **Figure S3**: The expression of NEAT1 in C28/I2 cells were detected by qPCR. **P < 0.01. n = 3/group.

**Additional file 5**. **Figure S4**: Quantification of Safranin O staining of C28/I2 cells with hsa_piR_019949 overexpression after transfected with NEAT1 (A) or NLRP3 (B). * indicated compared to Mimics NC group P< 0.01, ** indicated compared to Mimics NC group P < 0.01, ## indicated compared to pLVX-Vector+hsa_piR_019949 mimics group P < 0.01, n = 3/group.

## Data Availability

The datasets used during the current study are available from the corresponding author on reasonable request.
