# Supplementary figures and images for: PiRNA hsa_piR_019949 promotes chondrocyte anabolic metabolism by inhibiting the expression of lncRNA NEAT1
Source: J Orthop Surg Res. 2024 Jan 4;19:31. doi: 10.1186/s13018-023-04511-z (PMC10768105; doi:10.1186/s13018-023-04511-z)

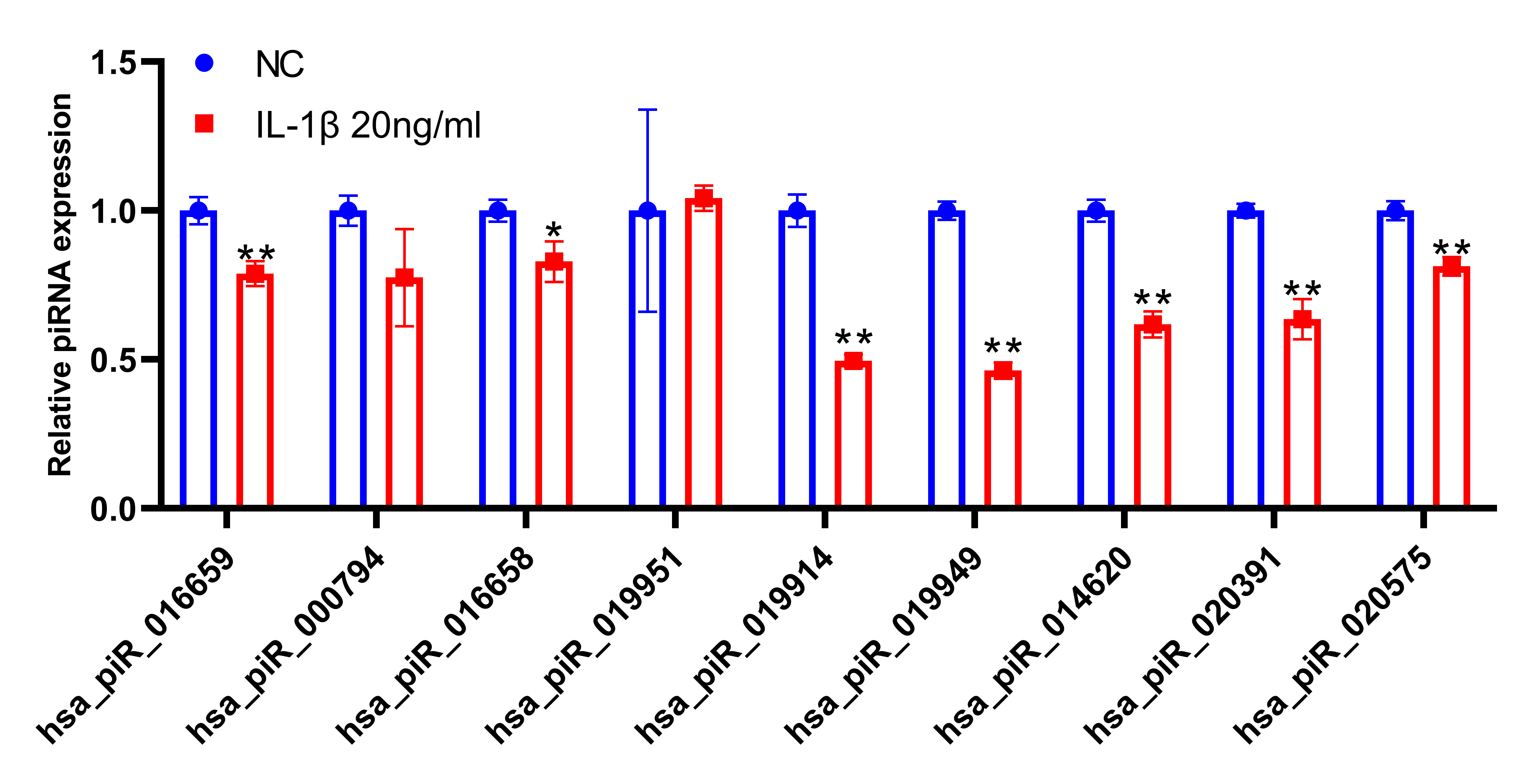

Supplement: Supplementary file 2 — Additional file 2. Figure S1: IL-1β regulated the expression of piRNA in C28/I2 cells. [file 13018_2023_4511_MOESM2_ESM.tif]

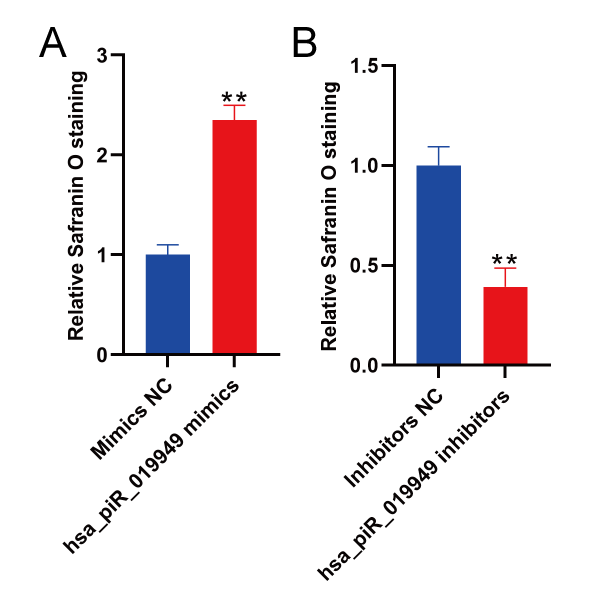

Supplement: Supplementary file 3 — Additional file 3. Figure S2: Quantification of Safranin O staining of C28/I2 cells with hsa_piR_019949 overexpression (A) or knockdown (B). **P < 0.01. n = 3/group. [file 13018_2023_4511_MOESM3_ESM.tif]

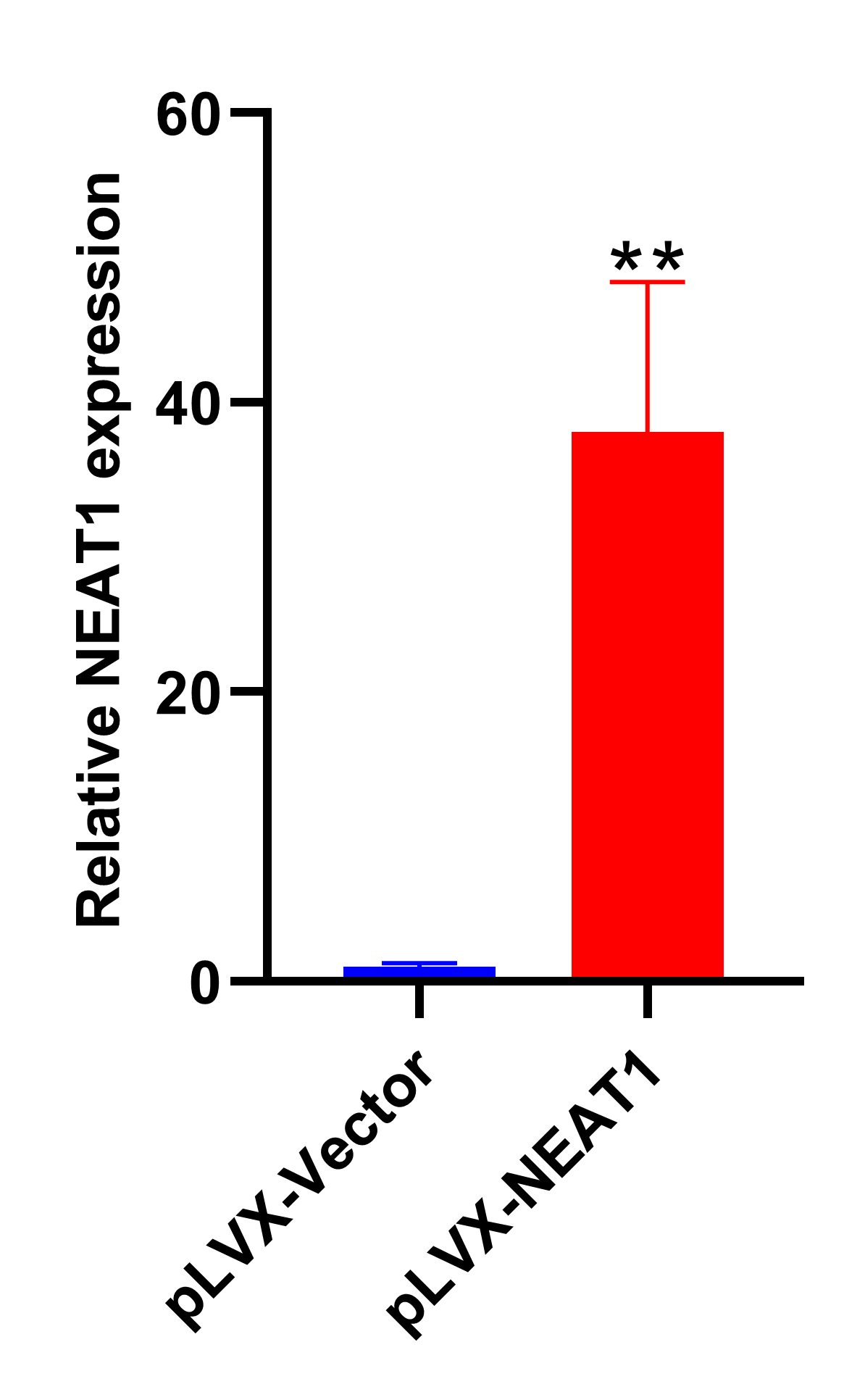

Supplement: Supplementary file 4 — Additional file 4. Figure S3: The expression of NEAT1 in C28/I2 cells were detected by qPCR. **P < 0.01. n = 3/group. [file 13018_2023_4511_MOESM4_ESM.tif]

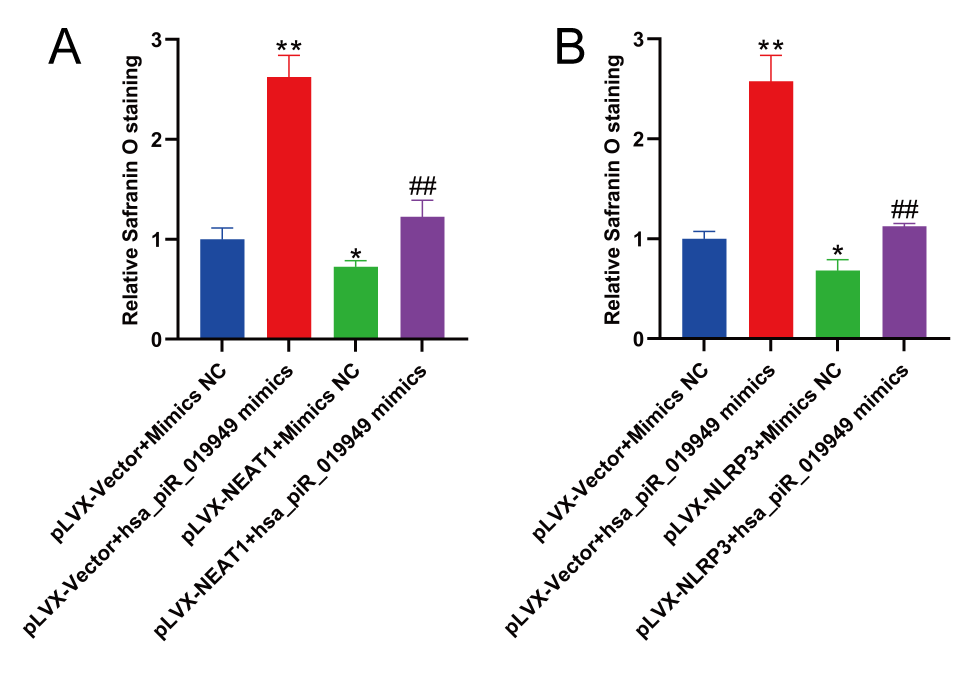

Supplement: Supplementary file 5 — Additional file 5. Figure S4: Quantification of Safranin O staining of C28/I2 cells with hsa_piR_019949 overexpression after transfected with NEAT1 (A) or NLRP3 (B). * indicated compared to Mimics NC group P< 0.01, ** indicated compared to Mimics NC group P < 0.01, ## indicated compared to pLVX-Vector+hsa_piR_019949 mimics group P < 0.01, n = 3/group. [file 13018_2023_4511_MOESM5_ESM.tif]
